# CRISPR-Cas: In Rekordzeit von der Grundlagenforschung zur Anwendung

**DOI:** 10.1007/s12268-020-1500-5

**Published:** 2020-11-25

**Authors:** Anita Marchfelder

**Affiliations:** Koordinatorin des DFG-Schwerpunkts SSP2141, Institut für Biologie II, Universität Ulm, Albert-Einstein-Allee 11, D-89081 Ulm, Deutschland

## Abstract

Den diesjährigen Nobelpreis für Chemie erhielten die beiden Forscherinnen Emmanuelle Charpentier und Jennifer Doudna für die Entdeckung des Genome Editing. Zum ersten Mal überhaupt in der Geschichte dieses Preises wurde er nur an Wissenschaftlerinnen verliehen.

■ Die Genschere CRISPR-Cas wurde auf der Basis des prokaryotischen Immunsystems entwickelt. Die Funktion des CRISPR-Cas-Systems wurde vor 13 Jahren im Rahmen mikrobiologischer Grundlagenforschung entdeckt; das System hilft Bakterien und Archaeen, sich gegen Angreifer zu wehren, z. B. Viren. Die zentralen Bestandteile des Systems sind die CRISPR-RNA und die Cas-Proteine. Für die Verteidigung werden die Informationen über die Angreifer in den CRISPR-Arrays gespeichert und machen die Zelle damit immun gegenüber wiederholten Angriffen eines Virus. Im Gegensatz zu den schon bekannten Verteidigungssystemen kann sich das CRISPR-Cas-System anpassen und die Immunität gegen die Angreifer vererben.

Bioinformatische Analysen haben gezeigt, dass die „Abwehrmaschine" in sehr vielen Varianten vorkommt, die sich hauptsächlich in der Art und Zusammensetzung der Cas-Proteine unterscheiden. Seit seiner Entdeckung werden die zahlreichen CRISPR-Cas-Versionen in mikrobiologischen Laboren rund um die Welt erforscht. Dieses Immunsystem ist extrem spannend für die Grundlagenforschung, da hier die Mechanismen der Reaktion untersucht und aufgeklärt werden, die neue Interaktionen zwischen Nukleinsäuren und Proteinen beschreiben. Überdies sind noch viele mikrobiologische Fragen zu klären: Wieso besitzen fast alle Archaeen, aber nur weniger als die Hälfte der Bakterien das CRISPR-Cas-System, warum ist es so ungleich verteilt? Was sind die Kosten des CRISPR-Cas-Systems für die Zelle, wie gefährlich ist die Autoimmunität? Wie werden Abwehr durch CRISPR-Cas und horizontaler Gentransfer ausgeglichen? Welche Funktionen hat das CRISPR-Cas-System zusätzlich zur Abwehr von Viren?

## Anwendung auch bei Covid-19-Tests

Für die Anwendung stellt das CRISPR-Cas-System eine riesige Schatztruhe dar, aus der neben der Genschere eine Fülle weiterer molekularbiologischen Werkzeugen entwickelt wurden, und auch in Zukunft sind hier noch viele neue Anwendungen zu erwarten. Die mannigfaltigen Varianten des Systems mit der Vielzahl an verschiedenen Cas-Proteinen geben eine breite Basis für die Anwendungsmöglichkeiten. Bisher hat sich die Methodenentwicklung hauptsächlich auf das Cas9-Protein konzentriert, aber immer mehr Anwendungen nutzen auch das Potenzial der zahlreichen anderen Cas-Proteine. Ein neuer Covid-19-Schnelltest nutzt z. B. die RNA-Abbau-Aktivität des Cas13-Proteins.

CRISPR-Cas ist nicht nur bei Pflanzen, Tieren und beim Menschen nutzbar, sondern genauso bei Bakterien und Archaeen. Es gibt viele Bakterien und Archaeen, die für die industrielle Fertigung von Lebensmitteln wichtig sind, bisher aber nur schlecht genetisch manipulierbar waren. Mithilfe der CRISPR-Cas-Werkzeuge lassen sich die Genome dieser Organismen jetzt auch verändern.

Die Entwicklung von CRISPR-Cas zeigt sehr deutlich, wie wichtig Grundlagenforschung ist, da hier innerhalb von wenigen Jahren – aufbauend auf den Erkenntnissen der mikrobiologischen Grundlagenforschung – extrem effiziente und vielfältige Anwendungsmöglichkeiten entwickelt wurden. Die Entdeckung des Genom Editing hat die Arbeit in den Laboren weltweit verändert. Sowohl für die Grundlagenforschung als auch für die Anwendung ist es ein sehr wichtiges Werkzeug.

In Deutschland wurde die Untersuchung dieses spannenden Abwehrsystems schon seit 2012 von der DFG im Rahmen einer Forschungsgruppe gefördert (2012–2017 FOR1680 *Unraveling the prokaryotic immune system*). Bei den Untersuchungen zeigte sich, dass das CRISPR-Cas-System neben der Verteidigungsfunktion auch andere Funktionen in der Zelle hat, z. B. bei der Biofilm-Bildung und der DNA-Reparatur. Diese Funktionen werden seit 2018 im Rahmen eines DFG-geförderten Schwerpunkts (SSP2141 *CRISPR-Cas functions beyond defence*; www.uni-ulm.de/nawi/crispr-cas-functions-beyond-defence-spp2141) untersucht. Bei beiden Konsortien war bzw. ist die Nobelpreisträgerin Emmanuelle Charpentier, die die Max-Planck-Forschungsstelle für die Wissenschaft der Pathogene in Berlin leitet, mit ihrer Gruppe assoziiertes Mitglied. ■

**Funding note:** Open Access funding enabled and organized by Projekt DEAL.
**Open Access:** Dieser Artikel wird unter der Creative Commons Namensnennung 4.0 International Lizenzveröffentlicht, welche die Nutzung, Vervielfältigung, Bearbeitung, Verbreitung und Wiedergabe in jeglichem Medium und Format erlaubt, sofern Sie den/die ursprünglichen Autor(en) und die Quelle ordnungsgemäß nennen, einen Link zur Creative Commons Lizenz beifügen und angeben, ob Änderungen vorgenommen wurden. Die in diesem Artikel enthaltenen Bilder und sonstiges Drittmaterial unterliegen ebenfalls der genannten Creative Commons Lizenz, sofern sich aus der Abbildungslegende nichts anderes ergibt. Sofern das betreffende Material nicht unter der genannten Creative Commons Lizenz steht und die betreffende Handlung nicht nach gesetzlichen Vorschriften erlaubt ist, ist für die oben aufgeführten Weiterverwendungen des Materials die Einwilligung des jeweiligen Rechteinhabers einzuholen. Weitere Details zur Lizenz entnehmen Sie bitte der Lizenzinformation auf http://creativecommons.org/licenses/by/4.0/deed.de.

**Korrespondenzadresse:**

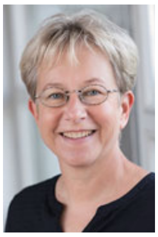

Prof. Dr. Anita Marchfelder
Koordinatorin des
DFG-Schwerpunkts SSP2141
Institut für Biologie II
Universität Ulm
Albert-Einstein-Allee 11
D-89081 Ulm
anita.marchfelder@uni-ulm.de
